# Computer Aided Diagnosis for Confocal Laser Endomicroscopy in Advanced Colorectal Adenocarcinoma

**DOI:** 10.1371/journal.pone.0154863

**Published:** 2016-05-04

**Authors:** Daniela Ştefănescu, Costin Streba, Elena Tatiana Cârţână, Adrian Săftoiu, Gabriel Gruionu, Lucian Gheorghe Gruionu

**Affiliations:** 1 Research Center of Gastroenterology and Hepatology Craiova, University of Medicine and Pharmacy, Craiova, Romania; 2 Endoscopy Department, Copenhagen University Hospital, Herlev, Denmark; 3 Department of Surgery, Massachusetts General Hospital and Harvard Medical School, Boston, United States of America; 4 Department of Engineering and Management of Technological Systems, Faculty of Mechanics, University of Craiova, Craiova, Romania; University of Jaén, SPAIN

## Abstract

**Introduction:**

Confocal laser endomicroscopy (CLE) is becoming a popular method for optical biopsy of digestive mucosa for both diagnostic and therapeutic procedures. Computer aided diagnosis of CLE images, using image processing and fractal analysis can be used to quantify the histological structures in the CLE generated images. The aim of this study is to develop an automatic diagnosis algorithm of colorectal cancer (CRC), based on fractal analysis and neural network modeling of the CLE-generated colon mucosa images.

**Materials and Methods:**

We retrospectively analyzed a series of 1035 artifact-free endomicroscopy images, obtained during CLE examinations from normal mucosa (356 images) and tumor regions (679 images). The images were processed using a computer aided diagnosis (CAD) medical imaging system in order to obtain an automatic diagnosis. The CAD application includes image reading and processing functions, a module for fractal analysis, grey-level co-occurrence matrix (GLCM) computation module, and a feature identification module based on the Marching Squares and linear interpolation methods. A two-layer neural network was trained to automatically interpret the imaging data and diagnose the pathological samples based on the fractal dimension and the characteristic features of the biological tissues.

**Results:**

Normal colon mucosa is characterized by regular polyhedral crypt structures whereas malignant colon mucosa is characterized by irregular and interrupted crypts, which can be diagnosed by CAD. For this purpose, seven geometric parameters were defined for each image: fractal dimension, lacunarity, contrast correlation, energy, homogeneity, and feature number. Of the seven parameters only contrast, homogeneity and feature number were significantly different between normal and cancer samples. Next, a two-layer feed forward neural network was used to train and automatically diagnose the malignant samples, based on the seven parameters tested. The neural network operations were cross-entropy with the results: training: 0.53, validation: 1.17, testing: 1.17, and percent error, resulting: training: 16.14, validation: 17.42, testing: 15.48. The diagnosis accuracy error was 15.5%.

**Conclusions:**

Computed aided diagnosis via fractal analysis of glandular structures can complement the traditional histological and minimally invasive imaging methods. A larger dataset from colorectal and other pathologies should be used to further validate the diagnostic power of the method.

## Introduction

Colorectal cancer (CRC) is the third most common cancer in the world, both in women and men, hence the need for a fast and accurate diagnosis [1]. Colonoscopy as a direct examination tool of the gastrointestinal tract, together with biopsy samples, is currently the gold standard for the diagnosis of neoplastic lesions and screening of the premalignant colorectal lesions [2, 3].

Local anatomical peculiarities can hinder successful biopsy of the colon lining; also, there is the risk of false negative biopsies by sampling tissue from areas which are wrongly diagnosed [4]. Recently, "optical biopsy" techniques have been developed to combine confocal microscopy with existing endoscopic equipment.

The potential of CLE has been explored in various diseases of the gastrointestinal tract. The ability to diagnose premalignant and malignant lesions is particularly important with direct implications in diagnosis and prognosis. High accuracy has been shown for CLE in detecting intraepithelial neoplasia, based on crypt architecture and vascular network pattern [5, 6]. We have previously used a dedicated confocal laser endomicroscope (eCLE), and the probe-based laser endomicroscopy system (pCLE) to visualize the intestinal mucosa at the microscopic level [7, 8].

Texture analysis of anatomical structures is a method used to interpret radiological and ultrasound images [9–11]. It has also been described as a potential method for diagnosing and assessing response to treatment in CT and MRI images in a variety of benign and malignant pathologies [10–13]. In a recent study that included a quantitative analysis of images recorded at colonoscopy with magnification, the homogeneity parameter was identified as a useful factor for the classification of colorectal lesions, showing significant differences between the different types of Kudo’s pit-pattern classification [14].

The aim of this study was to develop a computer aided diagnosis (CAD) algorithm for CRC, based on analyzing colon eCLE images, which can complement the existing immunohistological and imaging diagnosis methods.

## Material and Methods

This retrospective study was conducted on eCLE images from the database of the Research Center of Gastroenterology and Hepatology Craiova, University of Medicine and Pharmacy Craiova, Romania. A total number of 1035 images of normal or cancer colorectal mucosa (44.5±21.3 and 75.4±59.4 images per patient for normal and cancer respectively) were used for this analysis.

Before the eCLE procedure, all patients signed an informed consent form after being thoroughly explained the details of the study. The study was approved by the Committee of Ethics and Academic and Scientific Deontology, University of Medicine and Pharmacy of Craiova, Romania. The bowel preparation before the eCLE examination was done with 4 liters of Macrogol 4000 intestinal lavage solution (Fortrans).

### Confocal laser endomicroscopy procedure

Colon endomicroscopy investigation was performed using the EC-3870 CIFK, colonoscope (Pentax, Tokyo, Japan) as described before. This allowed conventional endoscopy and endomicroscopy examination in the same session. During the examination, the tissue is illuminated with a laser beam of 488 nm wavelength; the power exerted on the tissue surface can reach up to a maximum of 1 mW, offering the possibility to the examiner to adjust it in order to obtain optimum contrast and good quality images. The system provides a magnification of 1000X which enables observation of cellular and vascular details.

eCLE scanning allows targeted examination via the confocal lens which are placed slightly outside the distal tip of the endoscope. To obtain a histopathological diagnosis in real time, the examination was performed *in vivo*, after intravenous administration of 5 ml of 10% fluorescein. eCLE scanning was performed on a region of interest identified during colonoscopy. In order to maintain contact with the lens of the confocal microscope, but not deform the microanatomical structures, the colon lining was very gently aspirated. The resulting grey-scale 1024 x1024 pixels images were acquired at a rate of 0.8 images/second and were stored for further analysis (200–300 images per examination).

### Image analysis protocol and CAD parameter definition

The images generated during eCLE examinations were stored for offline processing. We have only used images without procedural artifacts such as bowel movement or slippage of particulate matter (bubbles, fecal debris). We processed the images using a computer aided diagnosis (CAD) module of a proprietary medical imaging system (NAVICAD) developed with the Matlab programming software (Matlab, The MathWorks Inc. USA).

The CAD application[15] includes three modules: anatomical feature identification module performed with functions from the MathWorks Image Processing Toolbox[16], the *isocontour* function based on Marching Squares and linear interpolation [17] and *polygeom* for feature perimeter calculation [18], the grey-level analysis based on the grey-level co-occurrence matrix (GLCM) in the *greycomatrix* function[19], and the fractal analysis module[20].

**Fig 1:**
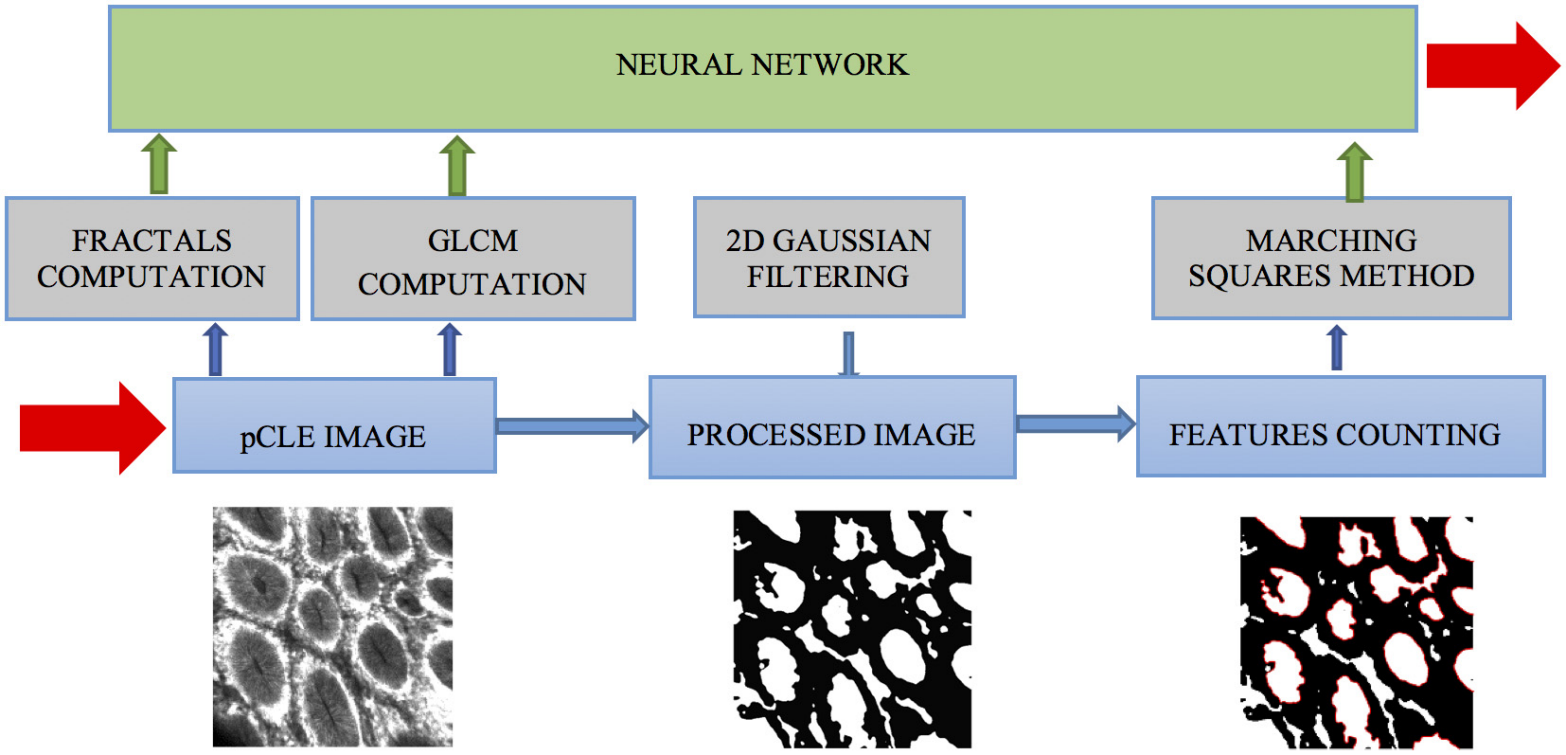
Diagram of the NAVICAD diagnosis application.

The application diagram is presented in Fig 1.

The first module of the application includes an algorithm that computes the fractal dimension and lacunarity for every image. The fractal dimension is a ratio providing a statistical index of complexity comparing how details in a pattern change with the scale at which they are measured, and also characterizes the space-filling capacity of a pattern. Lacunarity is a measure of how patterns fill space, with patterns having more or larger gaps having a higher lacunarity.

The second module uses the MATLAB function graycomatrix to compute the gray-level co-occurrence matrix (GLCM) for every image, which is considered a statistical method for examining texture that reflects the spatial relationship of pixels. The algorithm characterizes the texture of an image by calculating how often pairs of pixels with specific values and in a specified spatial relationship occur in an image, creating a matrix, and then extracting statistical measures like:

- Contrast—measurement of the local variations of image contrast,
- Correlation—measurement of the joint probability occurrence of a specified pixel pair,
- Energy—the sum of squared elements in the GLCM,
- Homogeneity—measurement of the closeness of the distribution of elements in the GLCM to the GLCM diagonal.

To identify specific anatomical features from the normal colon images, such as the glandular crypts, a third module was developed. In the first step, a Gaussian smoothing function is applied on the image to reduce the noise, and the result is converted to a binary image based on a threshold. The contour of anatomical features is identified using a Marching Square and linear interpolation algorithm, by which the ratio aria/perimeter is computed as a measure of feature roundness. The features with a ratio above an experimental determined value are counted for every image.

A normal colorectal mucosa tissue has many close to circular features (ratio above 4) compared to pathological tissues (ratio below 2) with chaotic, tortuous structure.

### Neural network implementation

A two-layer feed forward neural network was developed to diagnose images as normal or cancer based on the seven imaging parameters. The number of neurons in the pattern recognition hidden layer was 100 to balance accuracy vs. computational speed. We used two neurons for the output layer of the neural network, which corresponds with the normal vs. cancer diagnosis. The neural network was developed in Matlab (Matlab, The MathWorks Inc. USA) and used to solve a pattern-recognition problem in the decision module of the CAD application. The entire stack of image samples (both normal and cancer) were randomly divided in three categories: 725 for training, 155 for validating the training efficiency (stops training when not improving), and 155 images for testing the neural network diagnosis accuracy after it was trained and validated. The results of the neural network simulation are expressed in terms of the cross-entropy and decision accuracy error. The cross-entropy is an indicator of good classification of images, where lower values mean lower entropy and good classification and zero means no error. The decision accuracy error indicates the percentage of samples, which are misclassified, where 0 means no misclassifications and 100 indicates maximum misclassifications.

### Statistical analysis

The seven CAD parameter values are expressed as the mean ± standard deviation and are compared between normal and tumor values using a Student t-test with a p-value = 0.05 significance level in Microsoft Excel (Microsoft, Seattle, USA).

## Results

**Fig 2:**
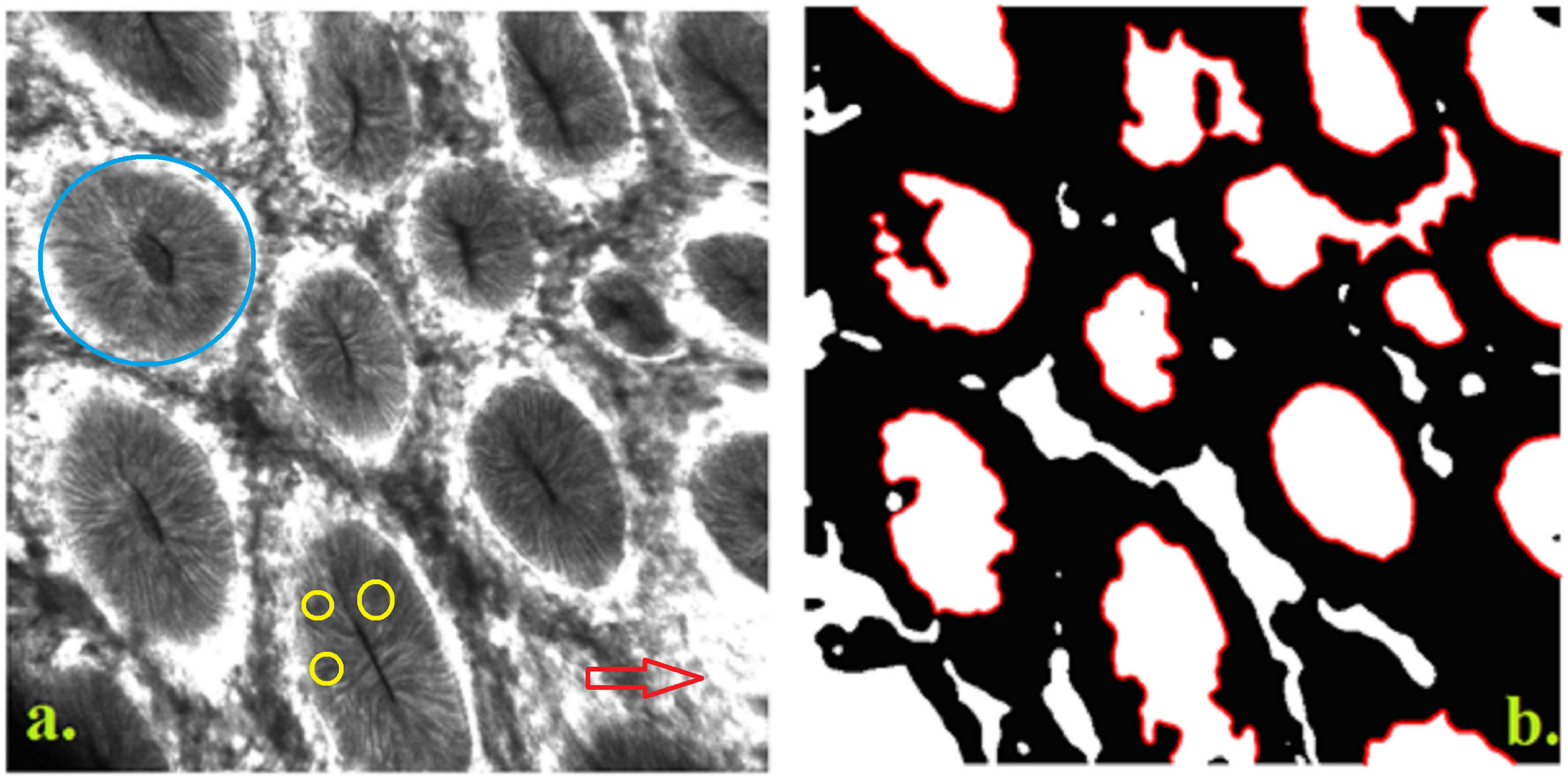
Normal colon mucosa. **a.** Normal colon mucosa with round shaped crypts (blue circle), situated at relatively equal distance one from another, dark goblet cells (yellow circles), and narrow and regular blood vessels surrounding the crypts (red arrows). **b.** Normal colon mucosa image processed: Fractal dimension = 1.732; Lacunarity = 0.13; Contrast = 0.26; Correlation = 0.97; Energy = 0.24; Homogeneity = 0.89; Features No = 14.

**Fig 3:**
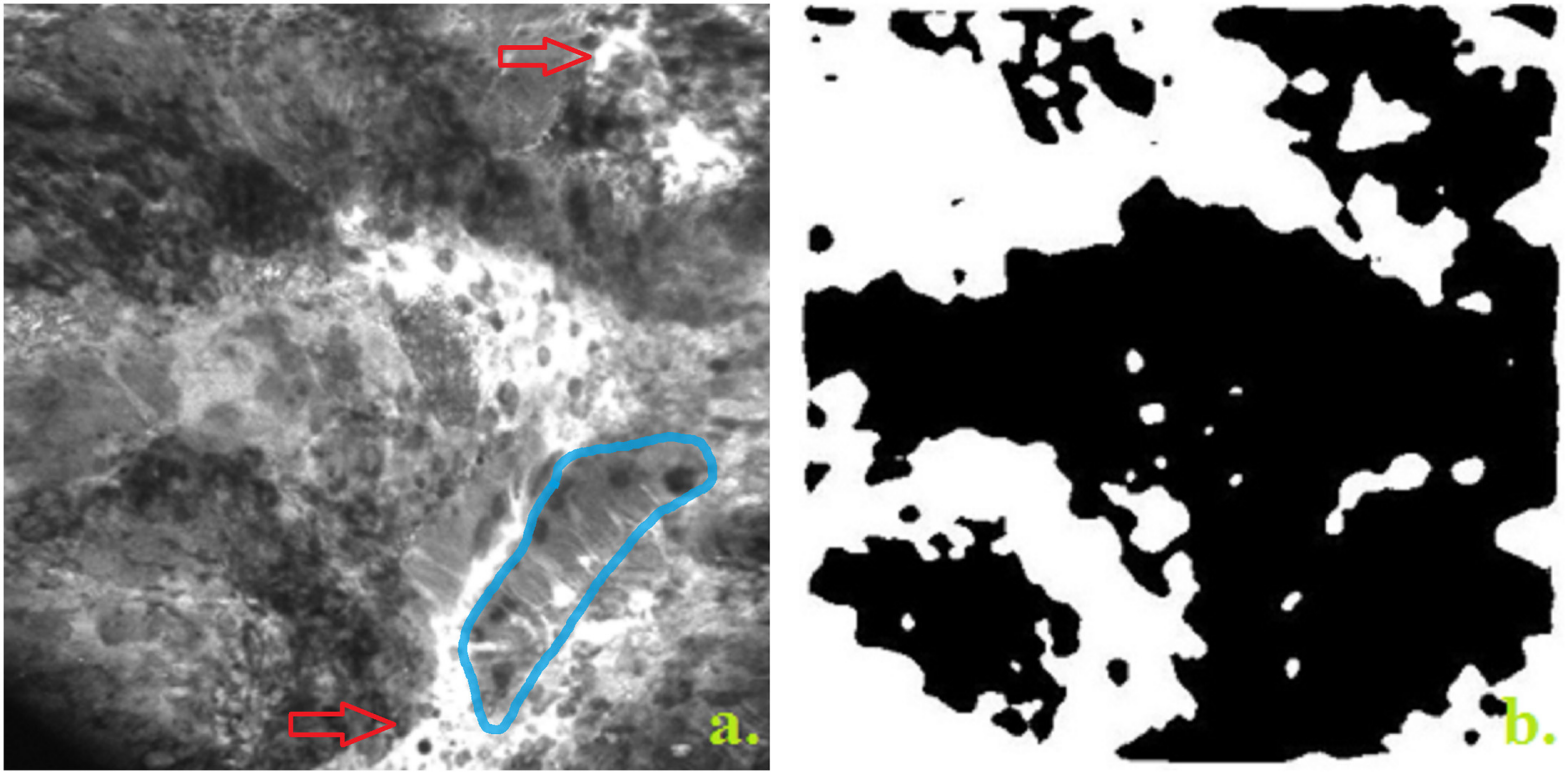
Adenocarcinoma. **a.** Adenocarcinoma with disorganized mucosa and lack of structure, crypts are elongated with irregularly thickened epithelium (blue), dilated and distorted blood vessels (red arrows). **b.** Adenocarcinoma image processed: Fractal dimension = 1.67; Lacunarity = 0.03; Contrast = 0.10; Correlation = 0.98; Energy = 0.15; Homogeneity = 0.95; Features No = 0.

eCLE imaging reveals the specific glandular crypts architecture of the normal mucosa (Fig 2). In contrast, the tumor mucosa shows disruption of the crypt hexagonal geometry which is hard to interpret by visual examination (Fig 3).

The seven parameters are presented in Table 1. While all are informative, only three parameters (contrast, homogeneity and feature number) are discriminative i.e. significantly different between normal and cancer samples.

**Table 1:** Average CAD parameter values ± standard deviation.

	Normal	Cancer
**Fractal Dimension**	1.88±0.05	1.91±0.04
**Lacunarity**	0.05±0.02	0.04±0.01
**Contrast**	0.28±0.04	0.24±0.03*
**Correlation**	0.93±0.02	0.93±0.03
**Energy**	0.14±0.02	0.17±0.04
**Homogeneity**	0.87±0.01	0.88±0.01*
**Feature No**	4.7±1.98	1.52±0.71*

Only contrast, homogeneity and feature number are significantly different than normal (*, p-value<0.05). The CAD fractal analysis simulation results for all patients are listed online [15].

The results of the neural network simulations are presented in Table 2. During the training phase the cross entropy was 0.53 and decision accuracy error was 15.5%.

**Table 2:** The neural network operation.

Neural Network Process	Sample No	Cross-Entropy	Decision Accuracy Error (%)
**Training**	725	0.53	16.14
**Validation**	155	1.17	17.42
**Testing**	155	1.17	15.48

## Discussion

CLE represents an important step in the evolution of gastrointestinal endoscopy, developed for better characterization of lesions identified during the examination [5]. Several clinical studies have shown that CLE has an increased accuracy for the *in vivo* histological diagnosis of colorectal cancer, based on real time interpretation of glandular crypts architecture as compared to histopathology alone [6]. However, this interpretation is dependent on the knowledge of histology and histopathology of the examining gastroenterologist. Furthermore, CLE yields a high number of images that have to be selected and interpreted, further limiting the real-time advantage of the method. Development of a fully automated computer-aided diagnosis algorithm can reduce the user-dependence on optical diagnosis obtained in real-time through CLE. This could prove especially useful for advanced colorectal carcinoma in order to improve accuracy and reduce inter-observer variability associated with qualitative CLE analysis of images.

Several virtual chromoendoscopy techniques have been proposed as red-flag techniques for the early detection of colorectal neoplasia in average-risk population. However, neither narrow-band imaging (NBI), nor i-SCAN had shown a significant difference as compared to high-definition bright field endoscopy [21]. Both NBI and CLE are useful for the differential diagnosis of colorectal neoplasia, although the learning curve could be long and requires training of the gastroenterologist based on pattern analysis. Although CAD algorithms have been developed and used with NBI images, there is little experience with CLE images, which provide higher magnification (and potentially higher accuracy) [22, 23].

Fractal analysis can be used as a measure of homogeneity, low values indicating decreasing homogeneity for image analysis [20, 24, 25]. We observed how the FD values of glandular structures were significantly different in the two studied groups, i.e. images of malignant tissue and normal mucosa. Not surprisingly, the average values of the CRC mucosa were higher compared to the normal group. Also we considered that a normal image has many close to circular features, compared to neoplastic images, and we noticed a larger average features number per image in normal cases than in cancer ones. One explanation may be that the invasive malignant neoplasms are histologically characterized by the destruction of the glandular crypts, which are replaced by malignant cells, as a result of clonal proliferation. A neoplastic tissue that is completely disorganized becomes a simplified, amorphous structure.

To improve the diagnosis accuracy we aimed to develop an objective method of interpretation based on several anatomical features. To our knowledge, the present study is the first report of fractal texture analysis being applied to the classification of colorectal lesions based on eCLE images of glandular structures. Thus, for each image, we determined a set of seven parameters to obtain sufficient information from which we sought to identify those relevant features that can distinguish classes of analyzed lesions. Out of those, only two parameters (contrast and feature number) were significantly different between normal and tumor samples. The other parameters (the fractal dimension, lacunarity, correlation, energy and homogeneity) were similar between groups. This pointed us to use a neural network analysis to study the combined effect of all parameters.

Fractal texture analysis provides information beyond what is visible at a simple inspection, without the need of additional images [26, 27]. Our study, even if carried out on a relatively small number of patients, keeps statistical significance due to the large number of images used for each studied group. We plan to continue this research on a larger number of patients, and further validate our methodology.

CLE could play a crucial diagnostic role for patients with CRC, by obtaining real-time virtual biopsies during colonoscopy. Additionally, the analysis of fractal images may be added to improve the quality of diagnosis. This approach could be used for a better selection of patients in clinical trials and careful monitoring of the chemotherapeutic agents effects in individualized therapies that are based on individual profiles created during these complex investigations.

## Conclusions

Computer aided diagnosis using fractal and other image feature parameters derived from CLE images can be successfully applied to characterize the morphometric structures of a normal colonic mucosa, and also to differentiate malignant areas. Using computational methods, we obtained seven key parameters which combined, could diagnose CRC in real-time. Future studies will investigate other pathologies which are characterized by histological feature differences.
